# Comparative chemical and biological study of essential oils and *n*-hexane extracts of *Thymus vulgaris* and *Thymus serpyllum* (Lamiaceae)

**DOI:** 10.1038/s41598-025-33660-w

**Published:** 2026-01-16

**Authors:** Mohamed M.M. AbdelRazek, Asmaa M. Atta, Nariman H. Kandil, Iriny E.G. Girgis, Rana T.O. Elsayed, Bassant R.M. Abdel-Latif, Asmaa E. Abdel-Halim, Shada G.I. Salama, Tag El-Din M. Ahmed, Mennat Allah S. AbdelRazek, Fady H. Foad, Rodaina M.S. Elsayed, Sara H.E. Aslan, Nada I.E. Badawy, Engy A. Farouk, Sara A. Omran, Khaled M. Darwish, Safaa A. El-Moghazy

**Affiliations:** 1 Department of Pharmacognosy, Faculty of Pharmacy, Badr University in Cairo (BUC), Cairo, 11829 Egypt; 2 Department of Pharmaceutical Chemistry, Faculty of Pharmacy, Badr University in Cairo (BUC), Badr, 11829 Cairo Egypt; 3 Internship Researcher, Faculty of Pharmacy, Badr University in Cairo (BUC), Cairo, 11829 Egypt; 4 Department of Medicinal Chemistry, Faculty of Pharmacy, Galala University, New Galala, 43713 Egypt; 5 Medicinal Chemistry Department, Faculty of Pharmacy, Suez Canal University, Ismailia, 41522 Egypt

**Keywords:** Thymus, Essential oil, *n*-hexane extract, Wound healing, GC-MS, Molecular docking, Biochemistry, Biotechnology, Chemical biology, Drug discovery, Plant sciences

## Abstract

This study presents a comparative chemical and biological evaluation of essential oils (EOs) and *n*-hexane extracts from *Thymus vulgaris* and *Thymus serpyllum* (Lamiaceae), alongside a reference commercial thyme oil. GC-MS analysis revealed significant differences in chemical composition among the samples, with *T. serpyllum* oil characterized by high thymol (74.47%) and caryophyllene (9.89%) content, while the commercial oil was dominated by carvacrol (80.37%). Cytotoxicity was assessed using the sulforhodamine B (SRB) assay on human skin fibroblast (HSF) cells to evaluate safety. Most samples exhibited low cytotoxicity (IC_50_ > 100 µg/mL), except for *T. serpyllum* oil, which showed strong cytotoxicity (IC_50_ = 18.48 µg/mL). Antidiabetic activity was evaluated via α-glucosidase and α-amylase inhibition assays. *T. serpyllum* oil demonstrated moderate α-glucosidase inhibition (IC_50_ = 536.9 ± 1.04 µg/mL), while other samples showed weak or negligible activity. Wound healing potential was assessed using an in vitro scratch assay on HSF cells. *T. serpyllum* oil at 0.05 µg/mL achieved complete wound closure within 72 h, outperforming other samples, including the commercial reference. Molecular docking of its major compounds (thymol, caryophyllene, α-selinene, and 3’,5’-dimethoxyacetophenone) revealed favorable binding affinities to key wound healing targets (collagenase, MMP-12, TGF-β), supporting the observed biological activity. Overall, *T. serpyllum* EO (TSV) exhibited superior wound healing capacity and moderate antidiabetic activity, with acceptable safety at low concentrations. These findings highlight its potential as a natural therapeutic agent and underscore the importance of standardizing thyme products for consistent pharmacological efficacy.

**Supplementary Information:**

The online version contains supplementary material available at 10.1038/s41598-025-33660-w.

## Introduction

 The genus *Thymus* (Lamiaceae) has served as a cornerstone herb in global traditional medicine, cosmetics, and culinary practices. The *thymus* genus commercial significance extends to pharmaceuticals, food preservation, and perfumery, driven by potent antimicrobial and anti-inflammatory properties^1,2^. The genus comprises 25 accepted species, with *Thymus vulgaris* (common thyme) and *Thymus serpyllum* (wild thyme) dominating research due to their rich phytochemistry and bioactivities^3^. Egypt’s distinct ethnomedicinal traditions further highlight *Thymus’s* versatility, where it is integrated into geriatric care, herbal teas, and natural therapeutics^4^.

The therapeutic power of *Thymus* species stems from their essential oils (EOs) and non-polar *n*-hexane extracts, which concentrate bioactive monoterpenes and phenolics. Key compounds include thymol, carvacrol, *p*-cymene, and γ-terpinene, responsible for broad-spectrum bioactivities^5^. *T. vulgaris* EOs exhibit high thymol and *p*-cymene content, with seasonal variations critically influencing yield and potency^6^. Conversely, *T. serpyllum* EOs are characterized by thymol (48–76%) and carvacrol (8–24%), displaying geographic compositional differences^7^. Non-polar *n*-hexane extracts are less commonly investigated but are vital for isolating lipophilic bioactives that drive pharmacological effects.

Diabetes mellitus is a multifactorial disorder often complicated by delayed wound healing, particularly in chronic cases such as diabetic foot ulcers^8^. *Thymus* species dual role in antidiabetic and wound healing activities has not been thoroughly studied with considering the cytotoxic activity. This research hypothesizes that bioactive compounds in *Thymus vulgaris* and *Thymus serpyllum* can target both glucose metabolism enzymes and wound healing pathways. Using in vitro and molecular docking approaches, the study seeks to validate their potential as natural agents for managing diabetic wounds. Carvacrol has been reported to improve diabetes by targeting key pathways such as insulin resistance, glucose uptake, and oxidative stress. Its antioxidant and anti-inflammatory effects further support its therapeutic potential^9^. Additionally, a study found that combining thymol and carvacrol produced better results than using either compound alone, suggesting a synergistic benefit^10^.

The wound healing process involves a tightly regulated sequence of events—hemostasis, inflammation, proliferation, and remodeling—driven by complex signaling networks and enzymatic activity. Among the critical molecular regulators are matrix metalloproteinases (MMPs) and growth factors. MMP-1 (collagenase) and MMP-12 (macrophage elastase) are zinc-dependent enzymes that degrade extracellular matrix (ECM) components, facilitating tissue remodeling. However, their overexpression can disrupt ECM integrity, leading to delayed or chronic wounds^11,12^. In contrast, transforming growth factor-beta (TGF-β) promotes wound repair by stimulating fibroblast activation, collagen synthesis, and modulation of inflammation^13^. Thus, targeting MMP-1, MMP-12, and TGF-β through molecular docking may offer a dual therapeutic approach—suppressing excessive ECM degradation while enhancing tissue regeneration.

The main goal of this study is to provide an integrated comparative analysis of the essential oils (EOs) and *n*-hexane extracts of *T. vulgaris* and *T. serpyllum*, linking their phytochemical profiles with their biological activities. This research presents the combined evaluation of the volatile and *n*-hexane extracts from two *Thymus* species, with a thorough correlation of the in vitro antidiabetic and wound healing activities with in silico results, which has not been reported in previous studies. This will offer deeper insight into how the chemotypic differences of *Thymus* plants influence their biological effectiveness and provide scientific guidance for the optimal use of *Thymus* species in wound healing and metabolic disorder treatments.

## Materials and methods

### Plant material and extraction

Fresh leaves of *Thymus vulgaris* and *Thymus serpyllum* were collected in January 2023 from a privately owned commercial farm belonging to botanist Ahmed AbdelRahman Mohamed, located in the Stella Heliopolis Compound near Shorouk City, Cairo, Egypt (GPS coordinates: 30°12′52.8″N, 31°40′57.4″E). The collection of plant materials was conducted with the full permission of the farm owner. No collection was made from wild populations, and the species are not listed as endangered or protected under national or international regulations and complies with institutional, national, and international guidelines, including the nternational Union for Conservation of Nature (IUCN) Policy Statement on Research Involving Species at Risk of Extinction and the Convention on the Trade in Endangered Species of Wild Fauna and Flora (CITES). Therefore, no special collection licenses or permits were required. Plants were collected by Engy A. Farouk (E.A.F.) and authenticated by the commercial farm owner, Botanist Ahmed AbdelRahman Mohamed, and validated by the Department of Pharmacognosy, Faculty of Pharmacy, Badr University in Cairo (BUC), Egypt. Voucher specimens have been deposited in the department’s herbarium under the accession codes BUC-PHG-TV-31 (*T. vulgaris*) and BUC-PHG-TS-32 (*T. serpyllum*). The samples (300 g) were subjected to steam distillation to extract essential oils (EOs), resulting in yields of 0.98% for *T. vulgaris* and 0.47% for *T. serpyllum*. Additionally, *n*-hexane maceration was employed (300 g) to obtain non-polar extracts, with yields of 2.27% for *T. vulgaris* and 1.2% for *T. serpyllum*. A commercial thyme oil product contains 1.5% *T. vulgaris* essential oil mixed with a plant-based fixed oil (Harraz, thyme essential oil catalogue code #1985, Egypt); was included in the comparative analysis as a market product.

### Chemical profiling

The chemical profiling of the essential oils (EOs) and *n*-hexane extracts was conducted using a GC-MS system (Shimadzu GCMS-QP2010 SE) equipped with an Rtx-5MS capillary column. One microliter of each sample was injected in split mode with a split ratio of 15:1, and helium was used as the carrier gas at a constant flow rate of 1.41 mL/min. The injection temperature was set at 250 °C, while the column oven temperature was initially held at 45 °C for 2 min, then increased at a rate of 5 °C/min to 300 °C, and held for 5 min. The ion source temperature was maintained at 200 °C, and the interface temperature at 280 °C. Mass spectra were recorded in electron ionization (EI) mode at 70 eV, with a scan range of m/z 35–500 and a scan speed of 1666 amu/sec. A solvent cut time of 3.00 min was applied. Compounds were identified by referencing their mass fragmentation patterns and Kovats retention indices, matched against the NIST (National Institute of Standards and Technology) mass spectral library^14^.

### Cytotoxic evaluation

Cytotoxicity was evaluated using the Sulforhodamine B (SRB) assay on human skin fibroblasts (HSF) obtained from the cell culture unit of Nawah Scientific Inc., (Mokatam, Cairo, Egypt) to evaluate the safety of the extracts^15,16^. The HSF cell line was originally sourced by Nawah Scientific from the American Type Culture Collection (ATCC) under the reference number BJ CRL-2522. Cells were cultured in DMEM (Dulbecco’s Modified Eagle Medium) supplemented with 10% heat-inactivated fetal bovine serum (FBS), 100 U/mL penicillin, and 100 µg/mL streptomycin under standard conditions (37 °C, 5% CO₂). Cells were seeded in 96-well plates at a density of 5 × 10³ cells/well and allowed to attach for 24 h. Test samples, including essential oils (EOs) and *n*-hexane extracts, were prepared in dimethyl sulfoxide (DMSO) and applied in serial concentrations ranging from 0.01 to 1000 µg/mL. After 72 h of incubation, cells were fixed with 10% trichloroacetic acid (TCA) for 1 h at 4 °C, then washed five times with distilled water and air-dried. Staining was performed using 0.4% (w/v) SRB solution for 10 min in the dark, followed by washing with 1% acetic acid and air drying overnight. Bound dye was solubilized with 10 mM TRIS base, and absorbance was measured at 540 nm using FLUOstar Omega microplate reader (BMG LABTECH, Germany). Cell viability was calculated as a percentage relative to untreated controls. Cytotoxicity (%) was then calculated as: Cytotoxicity (%) = 100 − Viability (%). Doxorubicin was used as a positive control. The IC_50_ values were calculated from dose–response curves, and Data were expressed as mean ± standard deviation (*n* = 3).

### Antidiabetic evaluation

The antidiabetic activity was assessed through α-glucosidase/α-amylase inhibition assays^15,17^. Samples were initially dissolved in dimethyl sulfoxide (DMSO) and diluted with methanol to ensure the final DMSO concentration did not exceed 1%. For the α-glucosidase assay, samples were tested at serial concentrations of 1000, 500, 250, 125, and 100 µg/mL, while for α-amylase, they were tested at 500 and 50 µg/mL. Acarbose was used as a standard control for both assays and tested at concentrations of 31.25–500 µg/mL for α-glucosidase and 0.1 and 1 µg/mL for α-amylase.

For the α-glucosidase assay, 25 µL of each sample or blank was incubated with 50 µL of α-glucosidase enzyme (0.6 U/mL) from Saccharomyces cerevisiae (Sigma-Aldrich, Cat. No. G5003) in 0.1 M phosphate buffer (pH 7) at 37 °C for 10 min. Then, 25 µL of 3 mM p-nitrophenyl-β-D-glucopyranoside (pNPG) substrate (Sigma-Aldrich, Cat. No. N7006) was added, and the mixture was incubated for 5 min at 37 °C.

For the α-amylase assay, 20 µL of each sample or blank was mixed with 140 µL of 50 mM phosphate buffer (0.9% NaCl, pH 7), followed by the addition of 20 µL of α-amylase enzyme (1 mg/mL) from porcine pancreas (Sigma-Aldrich, Cat. No. A3176). After incubation at 37 °C for 15 min, 20 µL of 2-chloro-4-nitrophenyl-α-D-maltotrioside (0.375 mM) substrate (Sigma-Aldrich, Cat. No. 93834) was added and incubated for another 10 min at 37 °C.

The absorbance was measured at 405 nm for both using a FLUOstar Omega microplate reader (BMG LABTECH, Germany). The percentage of enzyme inhibition for both assays was calculated using the following formula: % Inhibition = [(A_blank – A_sample) / A_blank] × 100.

where A_blank is the absorbance of the negative control (without inhibitor), and A_sample is the absorbance in the presence of the tested sample. Data were expressed as mean ± standard deviation (*n* = 3).

### Wound healing evaluation

The cell migration assay was used to assess the wound-healing potential of the samples using the human skin fibroblast (HSF) scratch model^11^. HSF cells were seeded at a density of 2 × 10⁵ cells/well in coated 12-well plates and cultured overnight in DMEM supplemented with 10% heat-inactivated fetal bovine serum (FBS), 100 U/mL penicillin, and 100 µg/mL streptomycin, at 37 °C in a humidified 5% CO₂ atmosphere. On the following day, a uniform horizontal scratch was introduced across the confluent monolayer using a sterile pipette tip. Detached cells were removed by gentle washing with PBS. Control wells were replenished with fresh medium, while treated wells received medium containing the test samples. Essential oils (EOs) and *n*-hexane extracts were tested at concentrations of 0.05 µg/mL and 0.5 µg/mL, respectively. These concentrations were selected based on the results of the sulforhodamine B (SRB) cytotoxicity assay, where IC_50_ values ranged from 18.48 µg/mL to > 100 µg/mL, ensuring that the tested doses were non-cytotoxic and appropriate for evaluating wound-healing activity. Cell migration was monitored at 0, 24, 48, 72, 96, and 120 h post-scratch. Wound width was calculated as the average distance between the two scratch edges at each time point. Quantitative measurements were performed using MII ImageView software version 3.7, and additional wound area analysis was conducted using Fiji-ImageJ software (NIH, Bethesda, MD). Microscopy images were measured but not captured during the experiment. However, the quantitative data obtained from the analysis provided reliable and reproducible measurements of cell migration and wound closure. Data were expressed as mean ± standard deviation (*n* = 3) to facilitate comparison of wound closure rates and assess variability across different treatment groups.

### Molecular Docking

**Fig. 1:**
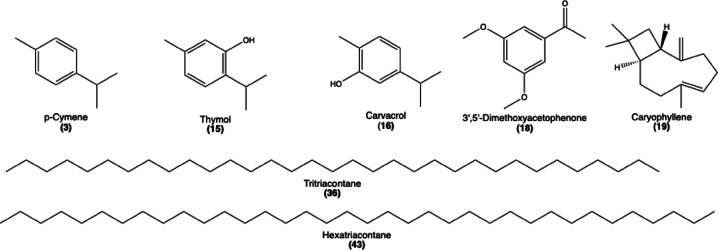
Chemical Structure of major constituents identified in different *Thymus* plants.

Using GC-MS analysis, 4 major compounds of *T. serpyllum* extract with peak areas surpassing 2% were identified for further exploration, including thymol, caryophyllene, α-Selinene, and 3’,5’-Dimethoxyacetophenone (Fig. 1). Molecular docking protocol was carried out using AutoDock Vina V.1.2.0 (Scripps Research, La Jolla, CA, United States) with standard preferences and parameters^18,19^. The X-ray crystal structures of the prospective wound healing targets: collagenase H complexed with N-aryl mercaptoacetamide-based inhibitor (PDB ID: 5O7E), matrix metalloproteinase-12 (MMP12) complexed with a beta hydroxy carboxylic acid (PDB ID: 2WO8), transforming growth factor-beta (TGF-β) in complex with N-(3-fluoropyridin-4-yl)-2-[6-(trifluoromethyl)pyridin-2-yl]-7 H-pyrrolo[2,3-d]pyrimidin-4-amine (PDB ID: 6B8Y) were obtained from protein data bank with reasonable resolution (≤ 2) and free R-value (≤ 0.25). The general docking procedures, including protein preparation (the removal of co-crystallized ligands, the elimination of water molecules, the addition of polar hydrogens, and the assignment of Kollman charge) and ligand preparation (modifying the charges and torsion angles) were performed using AutoDock Tools v.4.2.6^20^. The active site of each targeted enzyme is determined based on its corresponding co-crystalized ligand. To assess the reliability of the applied docking process, the co-crystalized ligands were separated and redocked once again to determine the root mean square deviation (RMSD). As expected, RMSD values of docked and co-crystalized ligand were 0.86, 0.85, and 1.43 for 5O7E, 2WO8, and 6B8Y, respectively, indicating the validity of the applied docking protocol. BIOVIA Discovery Studio (v21.1.0.20298) and PyMol V2.0.6 (Schrödinger, NY, USA) were used to visualize and analyze the molecular interactions between the docked compounds and the targeted enzymes.

### Statistical analysis

The biological activities of the *Thymus* extracts were assessed in triplicate to ensure reproducibility and reliability of the results. The data were analyzed using GraphPad Prism v10.1.1 and Microsoft Excel 365 to calculate means and standard deviations. Cytotoxicity data were expressed as mean ± SD of triplecate. One-way analysis of variance (ANOVA) followed by Tukey’s post hoc test was used to assess differences between the tested sample and the reference drug Doxorubicin (Dox) at each concentration. Significance levels were indicated as follows: *p* < 0.05 (*), *p* < 0.01 (**), and not significant (ns). For α-Glucosidase inhibitory activity of active samples > 1000 µg/mL, Dose-response curves were generated by plotting the percentage of α-glucosidase inhibition against the logarithm (log₁₀) of the sample concentrations. IC_50_ values were determined using nonlinear regression with a four-parameter logistic model. Differences between groups were evaluated using one-way ANOVA followed by Tukey’s post hoc test. A *p*-value of less than 0.05 was considered statistically significant. No inferential statistical tests were applied for wound healing activity, as presenting the data with descriptive statistics (mean ± SD) appropriately reflects the experimental outcomes.

## Results

### Comparative chemical profiling

A comparative chemical profiling of *Thymus vulgaris* (TV) and *Thymus serpyllum* (TS) was conducted using GC-MS analysis of their EOs and *n*-hexane extracts. The identified compounds are summarized in Table 1, indicating substantial qualitative and quantitative variations among the samples.

The essential oil (EO) of *T. serpyllum* (TSV) was predominantly characterized by carvacrol (80.37%), followed by thymol (9.32%) and *p*-cymene (10.3%). Other minor constituents included γ-terpinene (0.81%), trans-α-bergamotene (0.98%), α-selinene (3.37%), and caryophyllene (9.89%). However, the EO of *T. vulgaris* (TVV) was dominated by thymol (78.21%), carvacrol (3.69%), and linalool (2.5%). Notable minor constituents included eucalyptol (1.53%), α-phellandrene (0.15%), and terpinen-4-ol (0.85%). These results highlight that thymol and carvacrol are the principal phenolic monoterpenes in both species, yet their relative abundance varies significantly, with *T. serpyllum* showing a higher carvacrol-to-thymol ratio, while *T. vulgaris* showed the opposite trend.

Regarding the *n*-hexane extracts, *T. serpyllum* extract (TSH) showed a notable amount of tritriacontane (22.79%), hexatriacontane (44.2%), and 3-methylpentatriacontane (7.4%). Minor constituents included ethyl octacosyl ether (1.07%), octyl tetracosyl ether (1.38%), and urs-12-en-3-ol, acetate (0.8%). However, the *n*-hexane extract of *T. vulgaris* (TVH) was characterized by a broader diversity of long-chain aliphatic ethers and hydrocarbons, including hexatriacontane (2.36%), tritriacontane (26.53%), ethyl octacosyl ether (7.52%), and bis(2-ethylhexyl) phthalate (2.28%). Unique compounds such as 3-methyltritriacontane (2.04%) and heptyl hexacosyl ether (1.61%) were also detected in TVH but not in TSH.

Thymoquinone, a bioactive quinone derivative, was only identified in the *n*-hexane extracts, with a higher concentration in TVH (1.04%) than in TSH (0.39%). This suggests the potential pharmacological significance of the non-volatile fraction, particularly in *T. vulgaris*.

The compounds were identified in the reference thyme oil (REF) and TVV, while 97.86% and 98.42% were identified in TSV and TSH extracts, respectively. These findings demonstrate the complex phytochemical diversity between *T. vulgaris* and *T. serpyllum*, not only in their EOs but also in their *n*-hexane-soluble constituents. The differences in compound composition may underlie the reported variations in their therapeutic and aromatic properties. The variation in the major constituents showed in Fig. 1, which highlights the difference of chemical structure of majour compounds across the different thyme samples and emphasizes the chemotypic distinction between the two species (Table 2).

**Table 1:** Comparative GC-MS analysis of *n*-hexane extracts and EOs of *Thymus vulgaris* (TV) and *Thymus serpyllum* (TS).

SN	Compound	Chemical Class	Rt	M.F.	M.W.	RI _Cal_.	RI _Lit_.	Area %				
								REF	TSV	TVV	TSH	TVH
1	1-Octen-3-ol	Unsaturated aliphatic alcohol	8.48	C_8_H_16_O	128.21	976	976	–	–	0.63	–	–
2	α-Phellandrene	Monoterpene hydrocarbon	9.34	C_10_H_16_	136.23	1005	1005	–	0.15	–	–	–
3	*p*-Cymene	Aromatic monoterpene	9.80	C_10_H_14_	134.22	1020	1022	**10.3**	1.29	**2.27**	–	–
4	Eucalyptol	Monoterpene oxide	9.99	C_10_H_18_O	154.25	1026	1028	–	–	1.53	–	–
5	γ-Terpinene	Monoterpene hydrocarbon	10.86	C_10_H_16_	136.23	1054	1054	–	0.81	1.06	–	–
6	Bicyclo[3.1.0]hexan-2-ol, 2-methyl-5-(1-methylethyl)-, (1α,2β,5α)-	Monoterpene alcohol	11.12	C_10_H_18_O	154.25	1062	1057	–	–	1.89	–	–
7	Terpinolene	Monoterpene hydrocarbon	12.08	C_10_H_16_	136.23	1094	1094	–	–	0.49	–	–
8	Linalool	Monoterpene alcohol	12.15	C_10_H_18_O	154.25	1096	1097	–	–	2.5	–	–
9	Bicyclo[2.2.1]heptan-2-one, 1,7,7-trimethyl-, (1 S)-	Monoterpene ketone	13.50	C_10_H_16_O	152.23	1140	1139	–	–	0.84	–	–
10	Bicyclo[2.2.1]heptan-2-ol, 1,7,7-trimethyl-, (1 S-endo)-	Monoterpene alcohol	14.19	C_10_H_18_O	154.25	1162	1166	–	-	2.25	–	–
11	Terpinen-4-ol	Monoterpene alcohol	14.54	C_10_H_18_O	154.25	1174	1174	–	0.38	0.85	–	–
12	α-Terpineol	Monoterpene alcohol	14.96	C_10_H_18_O	154.25	1187	1189	–	–	0.23	–	–
13	Benzene, 2-methoxy-4-methyl-1-(1-methylethyl)-	Aromatic ether (phenylpropanoid)	16.25	C_11_H_16_O	164.24	1231	1231	–	–	0.34	–	–
14	Thymoquinone	Monoterpene quinone	16.70	C_10_H_12_O_2_	164.20	1248	1248	–	–	–	0.39	1.04
15	Thymol	Phenolic monoterpenoid	18.05	C_10_H_14_O	150.22	1293	1290	**9.32**	**74.47**	**78.21**	**10.9**	**44.6**
16	Carvacrol	Phenolic monoterpenoid	18.29	C_10_H_14_O	150.22	1301	1300	**80.37**	-	**3.69**	–	**4.27**
17	(1 S,2R,4R,7R)-4-Isopropyl-7-methyl-3,8-dioxatricyclo[5.1.0.02,4]octane	Oxygenated monoterpenoid	18.41	C_10_H_16_O_2_	168.23	1306	1306	–	–	–	–	0.67
18	3’,5’-Dimethoxyacetophenone	Aromatic ketone	21.22	C_10_H_12_O_3_	180.08	1407	1407	–	2.38		–	–
19	Caryophyllene	Sesquiterpene hydrocarbon	21.50	C_15_H_24_	204.35	1418	1418	–	**9.89**	1.62	–	–
20	trans-α-Bergamotene	Sesquiterpene hydrocarbon	21.88	C_15_H_24_	204.35	1432	1431	–	0.98	–	–	–
21	Humulene	Sesquiterpene hydrocarbon	22.40	C_15_H_24_	204.35	1452	1452	–	0.71	–	–	–
22	α-Selinene	Sesquiterpene hydrocarbon	23.27	C_15_H_24_	204.35	1485	1485	–	**3.37**	–	–	–
23	β-Selinene	Sesquiterpene hydrocarbon	23.50	C_15_H_24_	204.35	1494	1494	–	0.44	–	–	–
24	Caryophyllene oxide	Oxygenated sesquiterpene	25.73	C_15_H_24_O	220.35	1584	1583	–	1.06	1.6	0.49	–
25	Epicubenol	Sesquiterpene alcohol	26.49	C_15_H_26_O	222.37	1615	1616	–	0.39	–	–	–
26	τ-Cadinol	Sesquiterpene alcohol	27.09	C_15_H_26_O	222.37	1641	1640	–	0.72	–	–	–
27	(Z)-γ-Atlantone	Sesquiterpene ketone	28.35	C_15_H22O	218.33	1694	1698	–	0.23	–	–	-
28	(Z)-α-Atlantone	Sesquiterpene ketone	28.99	C_15_H_22_O	218.33	1721	1717	–	0.59	–	–	-
29	Bis(2-ethylhexyl) phthalate	Phthalate ester	44.46	C_24_H_38_O_4_	390.56	2546	2544	–	–	–	0.86	2.28
30	Ethyl hexacosyl ether	Long-chain aliphatic ether	49.54	C_28_H_58_O	410.76	2889	2891	–	–	–	0.38	2.51
31	Hexacosyl propyl ether	Long-chain aliphatic ether	50.91	C_29_H_60_O	424.79	2989	2982	–	–	–	-	0.79
32	Ethyl octacosyl ether	Long-chain aliphatic ether	52.24	C_30_H_62_O	438.81	3089	3086	–	–	–	1.07	7.52
33	2-Methylhentriacontane	Higher alkane	53.20	C_32_H_66_	450.87	3162	3160	–	–	–	–	0.7
34	Octacosyl propyl ether	Long-chain aliphatic ether	53.53	C_31_H_64_O	452.84	3188	3178	–	–	–	0.4	2.32
35	Octyl tetracosyl ether	Long-chain aliphatic ether	54.41	C_32_H_66_O	466.87	3250	3252	–	–	–	1.38	0.77
36	Tritriacontane	Higher alkane	55.04	C_33_H_68_	464.89	3295	3300	–	–	–	**22.79**	**26.53**
37	Nonyl tetracosyl ether	Long-chain aliphatic ether	55.96	C_33_H_68_O	480.89	3350	3350	–	–	–	0.28	–
38	Heptyl hexacosyl ether	Long-chain aliphatic ether	56.17	C_33_H_68_O	480.89	3363	3358	–	–	–	0.72	1.61
39	Tetratriacontane	Higher alkane	56.63	C_34_H_70_	478.92	3390	3400	–	–	–	**3.76**	–
40	Tritriacontane, 3-methyl-	Branched alkane	56.60	C_34_H_70_	478.92	3388	3380	–	–	–	-	2.04
41	Urs-12-en-3-ol, acetate, (3β)-	Triterpenoid ester	57.39	C_32_H_52_O_2_	468.75	3507	3486	–	–	–	0.8	–
42	3-Methylpentatriacontane	Branched alkane	57.80	C_36_H_74_	506.97	3536	3536	–	–	–	7.4	–
43	Hexatriacontane	Higher alkane	58.66	C_36_H_74_	506.97	3597	3600	–	–	–	**44.2**	2.36
44	Nonyl octacosyl ether	Long-chain aliphatic ether	60.79	C_37_H_76_O_2_	537.00	3742	3747	–	–	–	1.63	–
45	Butyl tetratriacontyl ether	Long-chain aliphatic ether	62.41	C_38_H_78_O	551.03	3854	3892	–	–	–	0.99	–

REF: Reference market product of thyme oil, TSV: *Thymus serpyllum* essential oil (EO), TVV: *Thymus vulgaris* EO, TSH: *Thymus serpyllum n*-hexane extract, TVH: *Thymus vulgaris n*-hexane extract. Compounds were identified using Kovet’s indexes and mass spectrum fragmentation chromatograms in the NIST library with a range of ± 5.

**Table 2:** Chemical class composition.

Chemical Class	Area percentage (%) of identified compounds				
	REF	TSV	TVV	TSH	TVH
Monoterpenes hydrocarbons	10.3	2.25	3.82	–	–
Oxygenated monoterpenes	–	0.38	12.33	–	–
Phenolic & quinone monoterpenes	89.69	74.47	81.90	11.29	49.91
Aromatic/phenylpropanoid compounds	–	2.38	0.34	–	–
Sesquiterpenes hydrocarbons	–	15.39	1.62	–	–
Oxygenated sesquiterpenes	–	3.00	1.60	0.49	–
Phthalate esters	–	–	–	0.86	2.28
Long-chain aliphatic ethers	–	–	–	6.87	18.52
Alkanes (linear & branched)	–	–	–	78.91	32.40
Triterpenoid esters	–	–	–	0.80	–
Total Compounds Identified (%)	100	97.86	100	98.42	100

REF: Reference market product of thyme oil, TSV: *Thymus serpyllum* essential oil (EO), TVV: *Thymus vulgaris* EO, TSH: *Thymus serpyllum n*-hexane extract, TVH: *Thymus vulgaris n*-hexane extract.

### Cytotoxic evaluation

**Fig. 2:**
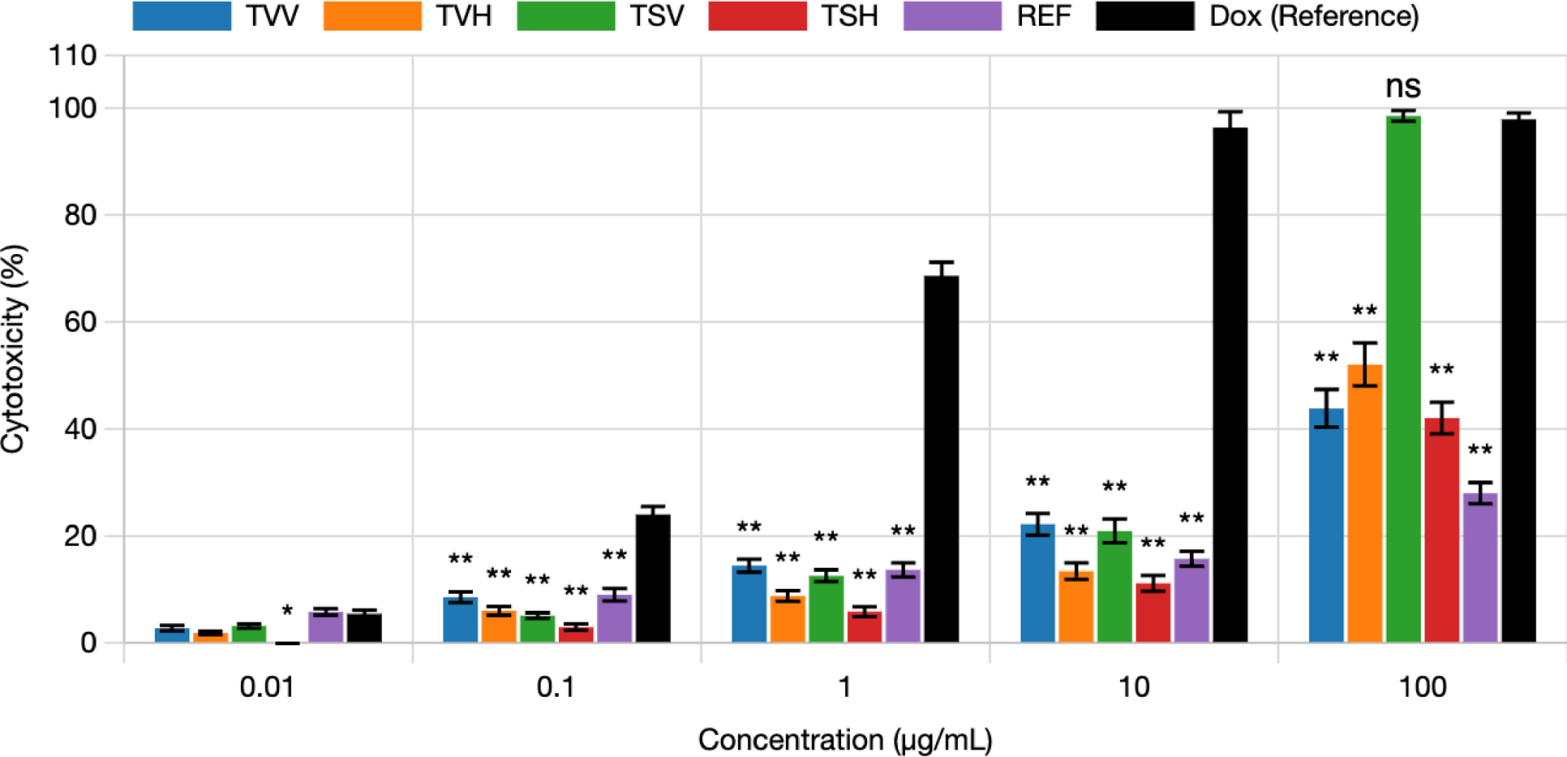
Dose response chart of cytotoxic activity using SRB assay. TVV: *Thymus vulgaris* essential oil (EO), TVH: *Thymus vulgaris n*-hexane extract, REF: Reference market product of thyme oil, TSV: *Thymus serpyllum* EO, TSH: *Thymus serpyllum n*-hexane extract, Dox.: Doxorubicin as a reference drug. Bars represent mean values of three replicates. Asterisks indicate statistical significance compared to Dox at the same concentration (*p < 0.05, **p < 0.01, ns = not significant; one-way ANOVA with Tukey’s post hoc test). Error bars represent ± standard deviation.

The cytotoxicity of the tested *Thymus* extracts and oils was evaluated against a human normal fibroblast cell line using the sulforhodamine B (SRB) assay, to assess their safety by measuring potential toxicity against normal cells. Among the tested samples, *T. serpyllum* EO (TSV) demonstrated the highest cytotoxicity, with an IC_50_ value of 18.48 µg/mL, indicating a considerable toxic effect on normal fibroblasts. *T. vulgaris n*-hexane extract (TVH) showed moderate cytotoxicity, with an IC_50_ of 94.49 µg/mL. In contrast, *T. vulgaris* EO (TVV), *T. serpyllum n*-hexane extract (TSH), and the reference thyme oil product (REF) exhibited low or negligible toxicity, with IC_50_ values greater than 100 µg/mL, suggesting a more favorable safety profile. As expected, the reference anticancer drug doxorubicin (Dox.) displayed strong cytotoxicity, with an IC_50_ value of 0.38 µg/mL. These results indicate that most of the tested *Thymus* samples, except for TSV and to a lesser extent TVH, are relatively safe for normal human fibroblast cells, supporting their potential for further development with minimal toxicity concerns (Table 3, Fig. 2).

**Table 3:** Cytotoxic IC_50_ using SRB assay.

Sample	IC_50_ (µg/mL)
TVV	> 100
TVH	94.49
TSV	18.48
TSH	> 100
REF	> 100
Dox.	0.38

TVV: *Thymus vulgaris* essential oil (EO), TVH: *Thymus vulgaris n*-hexane extract, REF: Reference market product of thyme oil, TSV: *Thymus serpyllum* EO, TSH: *Thymus serpyllum n*-hexane extract, Dox.: Doxorubicin as a reference drug.

### Antidiabetic activity

**Fig. 3:**
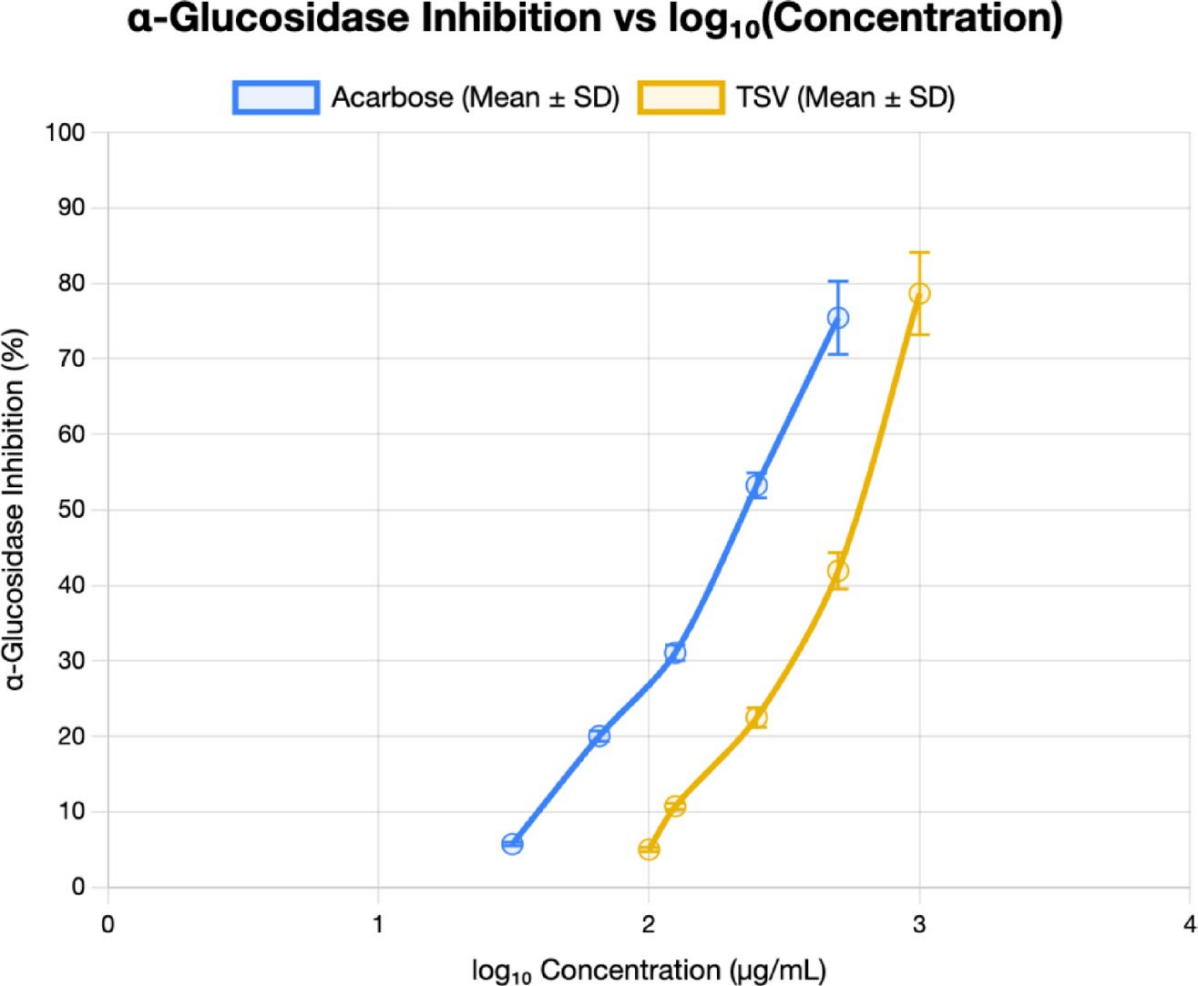
IC_50_ of *α*-Glucosidase inhibitory activity of *Thymus serpyllum* EO (TSV) compared to acarbose.

The antidiabetic activity of various *Thymus n*-hexane extracts and essential oils was evaluated using enzyme inhibitory assays targeting α-glucosidase and α-amylase. The results, expressed as IC_50_ values (µg/mL), revealed that *T. serpyllum* EO (TSV) exhibited moderate inhibitory activity against α-glucosidase with an IC_50_ value of 536.9 ± 1.040 µg/mL, while showing no significant inhibition against α-amylase (IC_50_ > 500 µg/mL). However, *T. vulgaris* EO (TVV), the reference thyme oil product (REF), *T. serpyllum n*-hexane extract (TSH), and *T. vulgaris n*-hexane extract (TVH) all demonstrated weak or negligible inhibitory effects against both enzymes, with IC_50_ values exceeding 1000 µg/mL for α-glucosidase and 500 µg/mL for α-amylase. Acarbose, the standard antidiabetic drug used as a positive control, exhibited potent inhibitory effects with IC_50_ values of 219.6 ± 1.030 µg/mL for α-glucosidase and 6.47 ± 1.034 µg/mL for α-amylase. These findings suggest that among the tested samples, only *T. serpyllum* EO (TSV) demonstrated moderate α-glucosidase inhibition, though it was markedly less effective than acarbose (Table 4, Fig. 3).

**Table 4:** Antidiabetic activity using enzyme inhibitory assay.

Sample	IC_50_ (µg/mL)	
	α-glucosidase	α-amylase
TVV	> 1000	> 500
TSV	536.9 ± 1.040	> 500
REF	> 1000	> 500
TSH	> 1000	> 500
TVH	> 1000	> 500
Acarbose	219.6 ± 1.030	6.47 ± 1.034

REF: Reference market product of thyme oil, TSV: *Thymus serpyllum* essential oil (EO), TVV: *Thymus vulgaris* EO, TSH: *Thymus serpyllum n*-hexane extract, TVH: *Thymus vulgaris n*-hexane extract, Acarbose as a reference drug. Data are presented as mean ± standard deviation (SD).

Data are presented as mean ± standard deviation (SD). Error bars represent ± standard deviation. IC_50_ Acarbose = 219.6 ± 1.030 µg/mL (log₁₀ = 2.34), TSV = 536.9 ± 1.040 µg/mL (log₁₀ =2.73).

### Wound healing evaluation

**Fig. 4:**
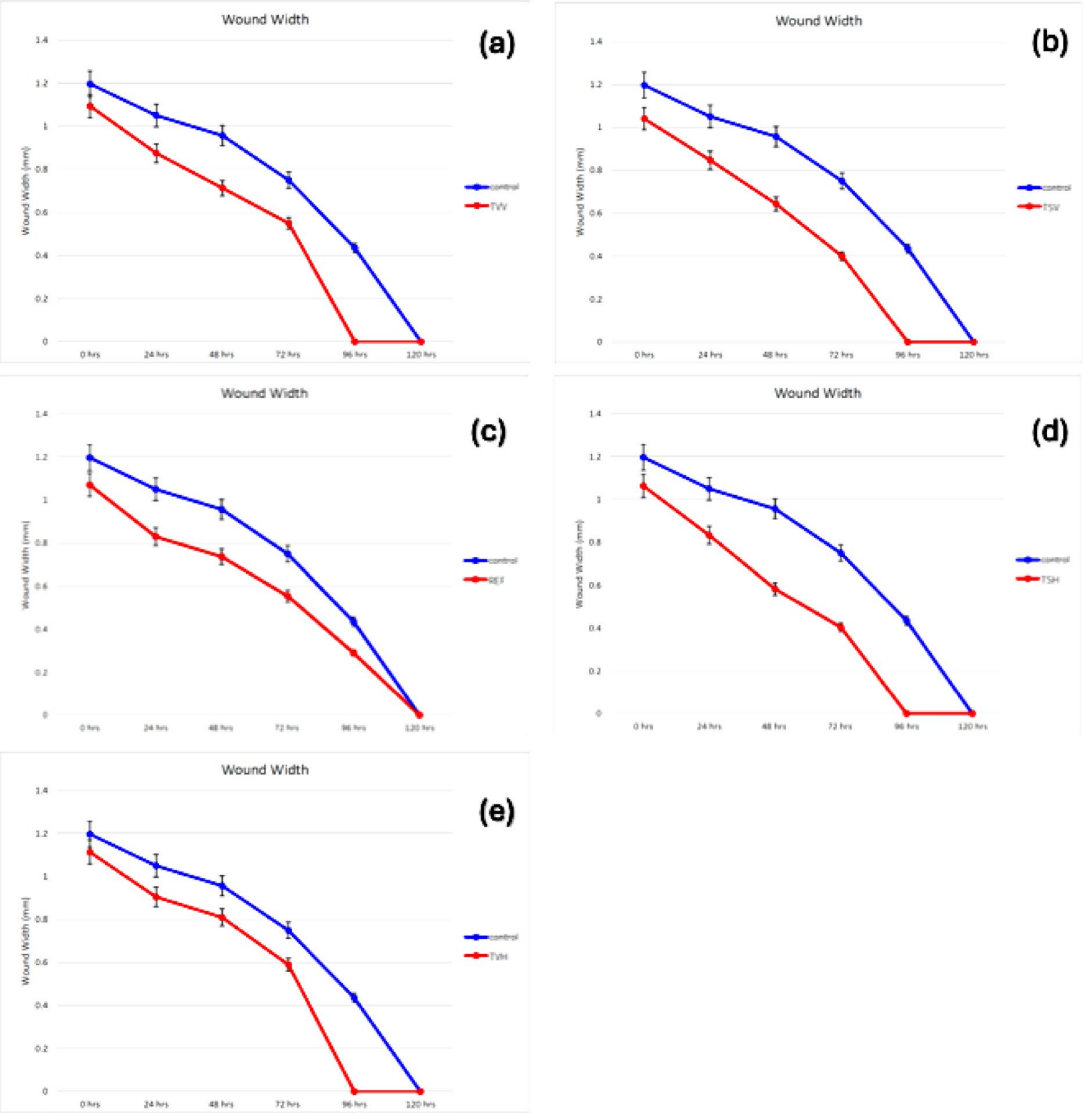
Dose response curve of cell migration assay for wound healing activity at different time intervals. (**a**) TVV: *Thymus vulgaris* essential oil (EO), (**b**) TVH: *Thymus vulgaris n*-hexane extract, (**c**) REF: Reference market product of thyme oil, (**d**) TSV: *Thymus serpyllum* EO, (**e**) TSH: *Thymus serpyllum n*-hexane extract. Data are presented as mean ± standard deviation (SD).

The wound healing activity of the tested *Thymus* extracts and oils was evaluated using in vitro cell migration assay on a human normal fibroblast cell line, aiming to assess their potential in promoting tissue regeneration and repair. The results demonstrated that *T. serpyllum* EO (TSV) at a concentration of 0.05 µg/mL exhibited the most potent wound healing effect, achieving complete wound closure (100%) within 72 h. In comparison, the untreated control group showed significantly slower healing, with a wound width of 0.75 μm remaining at 72 h and incomplete closure (0.435 μm) even after 96 h. Other samples, including *T. vulgaris* EO (TVV), the reference thyme oil product (REF), *T. serpyllum n*-hexane extract (TSH), and *T. vulgaris n*-hexane extract (TVH), showed progressive wound healing, with complete closure occurring between 96 and 120 h depending on the sample. Notably, TVV and REF (both at 0.05 µg/mL) achieved full closure by 120 h, while TSH and TVH (at 0.5 µg/mL) reached closure by 96 h. These findings suggest that TSV markedly enhances fibroblast migration and wound closure in vitro, highlighting its potential as a natural agent for promoting wound healing (Table 5, Fig. 4).

**Table 5:** Wound healing activity using cell migration assay.

Sample	Conc. µg/mL	Wound width (µm)					
		0 h	24 h	48 h	72 h	96 h	120 h
TVV	0.05	1.09 ± 0.072	0.88 ± 0.021	0.71 ± 0.121	0.55 ± 0.269	0	0
TSV	0.05	1.04 ± 0.014	0.85 ± 0.025	0.64 ± 0.040	0	0	0
REF	0.05	1.07 ± 0.017	0.83 ± 0.017	0.74 ± 0.023	0.56 ± 0.050	0.29 ± 0.0707	0
TSH	0.5	1.06 ± 0.153	0.83 ± 0.208	0.58 ± 0.021	0.41 ± 0.212	0	0
TVH	0.5	1.11 ± 0.208	0.91 ± 0.120	0.81 ± 0.099	0.59 ± 0.014	0	0
Control	-	1.20 ± 0.006	1.05 ± 0.036	0.96 ± 0.045	0.75 ± 0.044	0.435 ± 0.007	0

REF: Reference market product of thyme oil, TSV: *Thymus serpyllum* essential oil (EO), TVV: *Thymus vulgaris* EO, TSH: *Thymus serpyllum n*-hexane extract, TVH: *Thymus vulgaris n*-hexane extract. Data are presented as mean ± standard deviation (SD).

### Molecular Docking

As *T. serpyllum* extract exhibited the superior wound healing potential, the molecular docking analysis has been employed to get more insight into the binding affinities and molecular binding interactions of its major compounds with the key targets related to wound healing (collagenase, MMP12, and TGF-β). Thus, the four major identified compounds were docked into the active site of three target proteins, taking the co-crystalized ligands as a template. Docking studies revealed that almost all compounds demonstrated acceptable binding affinities against the three targets (Table 6). Amongst all identified compounds, thymol and 3’,5’-dimethoxyacetophenone achieved the superior docking scores against the three targets. Both compounds were just inferior to the redocked co-crystallized ligands of each respective biotarget.

**Table 6:** Docking scores (in kcal/mol) of the major identified compounds from *T. serpyllum* against collagenase (PDB: 5O7E), MMP12 (PDB ID: 2WO8), and TGF-β (PDB ID: 6B8Y).

Compound name	Docking scores (kcal/mol)		
	Collagenase (PDB: 5O7E)	MMP12 (PDB: 2WO8)	TGF-β (PDB: 6B8Y)
Thymol	-5.2	-5.8	-5.7
3’,5’-Dimethoxy acetophenone	-5.6	-5.3	-5.2
Caryophyllene	-4.6	-4.3	-4.8
α-Selinene	-4.8	-4.9	-4.4
Co-crystalized ligand	-6.1	-8.1	-6.2

**Fig. 5:**
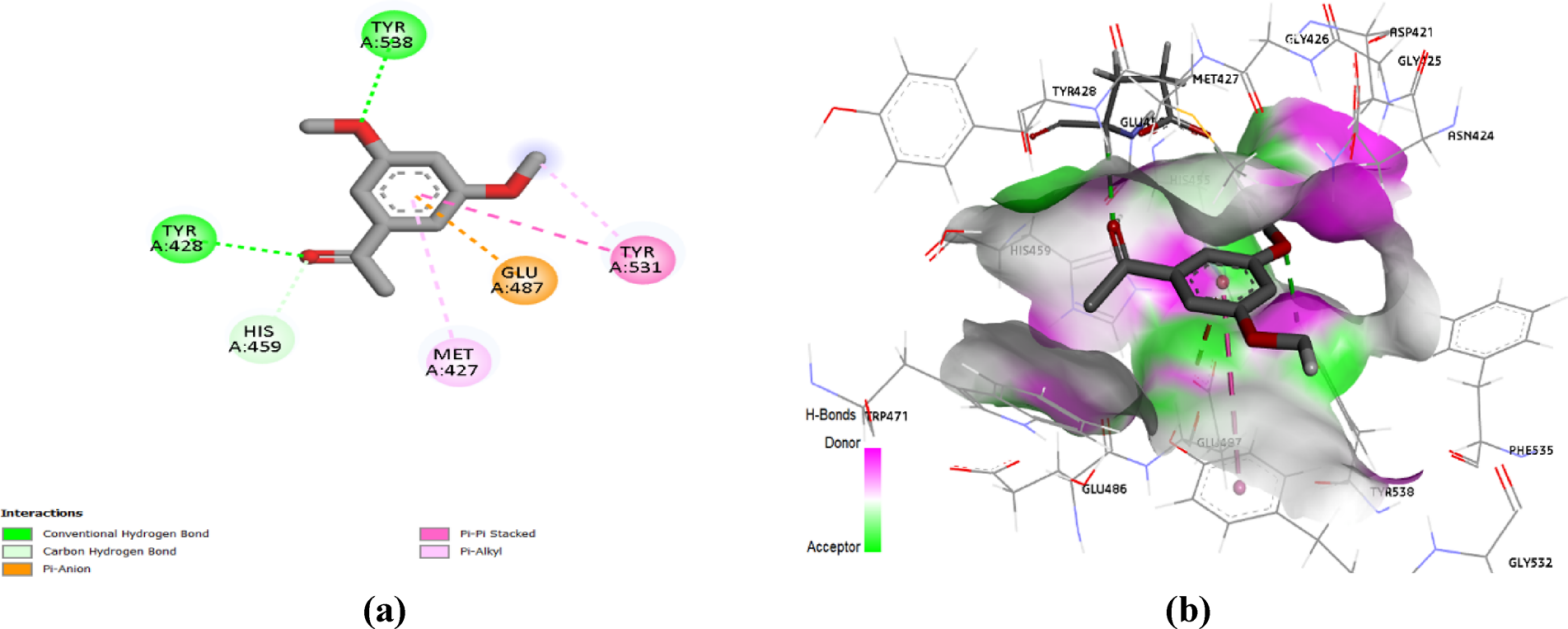
(**a**) Two-dimensional interactions and (**b**) three-dimensional interactions of 3’,5’-dimethoxyacetophenone with the active site of collagenase target enzyme (PDB:5O7E).

**Fig. 6:**
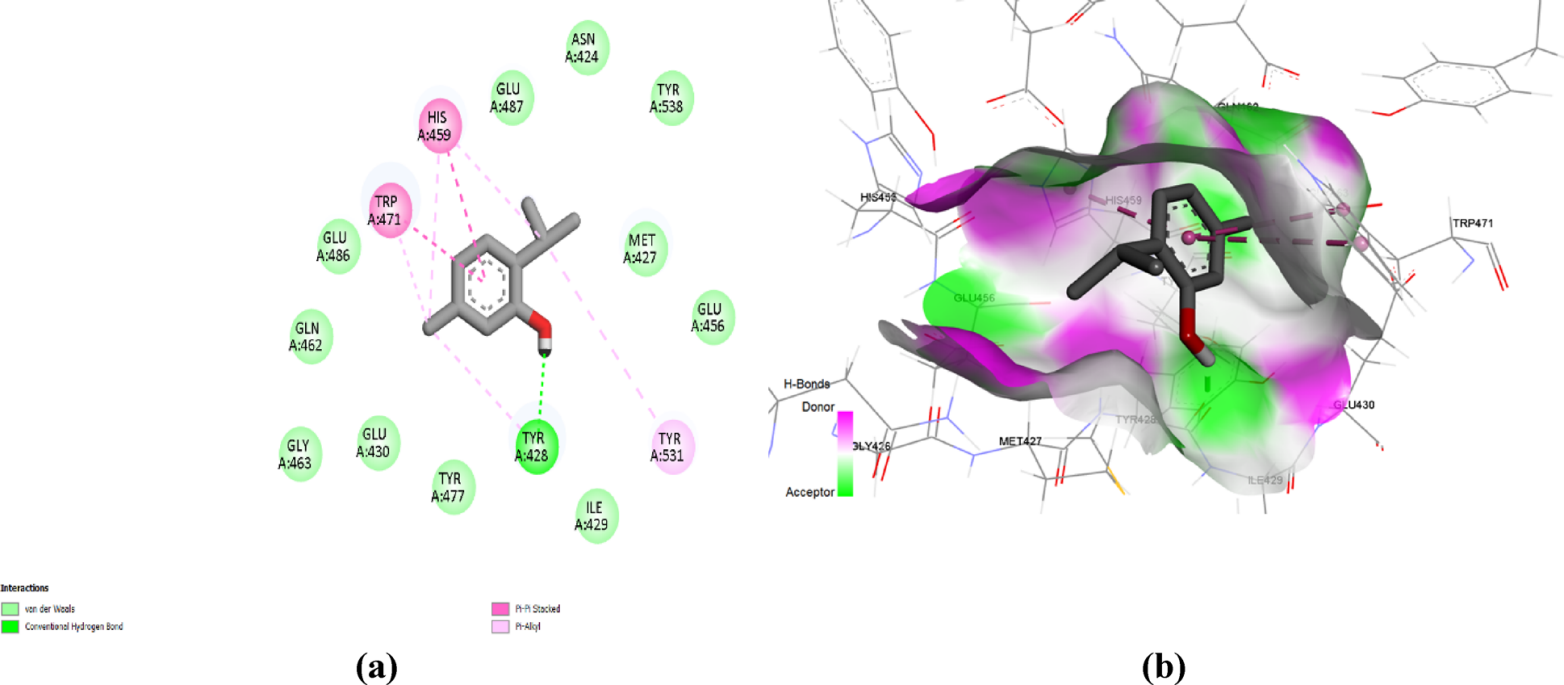
(**a**) Two-dimensional interactions and (**b**) three-dimensional interactions of thymol with the active site of collagenase target enzyme (PDB:5O7E).

In the docking of collagenase, 3’,5’-dimethoxyacetophenone and thymol demonstrated the superior pose scores of -5.6 and − 5.2 Kcal/ Mol, respectively. In a comparable orientation fashion of the co-crystallized ligand, both 3’,5’-dimethoxyacetophenone and thymol were anchored at the active site cleft settled horizontally in between the lower carboxy- and upper amino-terminal subdomains of the Peptidase domain. However, the co-crystallized ligand showed a more extended conformation in relation to that of the two natural docked compounds. The increased size of the co-crystallized ligand can highly rationalize its higher docking score (‒6.1 kcal/mol) as compared to those of the natural compounds. Figure 5 presented that 3’,5’-dimethoxyacetophenone showed interactions with six amino acid residues, in which two were conventional hydrogen bonds (Trp428 and Trp538), one Pi-anion interaction (Glu487), one carbon-hydrogen bond (His459), two Pi-alkyl interactions (Trp531, Met427), and one Pi-Pi stacking interaction (Tyr531). While thymol interacted with Trp428 through a conventional hydrogen bond and with Tyr531, Trp471, and His459 through a network of hydrophobic interactions (Fig. 6). Notably, both compounds bind to the His459 residue that is included within the zinc-binding motif (HEXXH), suggesting potential interference with the enzyme’s catalysis by both compounds. The HEXXH is at the amino-terminal subdomain α-helix, providing the enzyme with relevant stability and coordination for its zinc atom via the double Histidine residues (His455 and His459), while the acid/base residue, Glu456, directly participates in the enzyme’s active machinery^21^. Besides the HEXXH motif, the Glu487 residue on the glutamate helix of the carboxy-terminal subdomain further participates in zinc atom coordination. Interestingly, the 3’,5’-dimethoxyacetophenone was aligned with the redocked co-crystallized ligand as both depicted inter-molecular interaction towards the Glu487 side chain. The latter compound-Glu487 interaction would rationalize its higher docking score and, in turn, the better biological significance of 3’,5’-dimethoxyacetophenone in relation to Thymol.

**Fig. 7:**
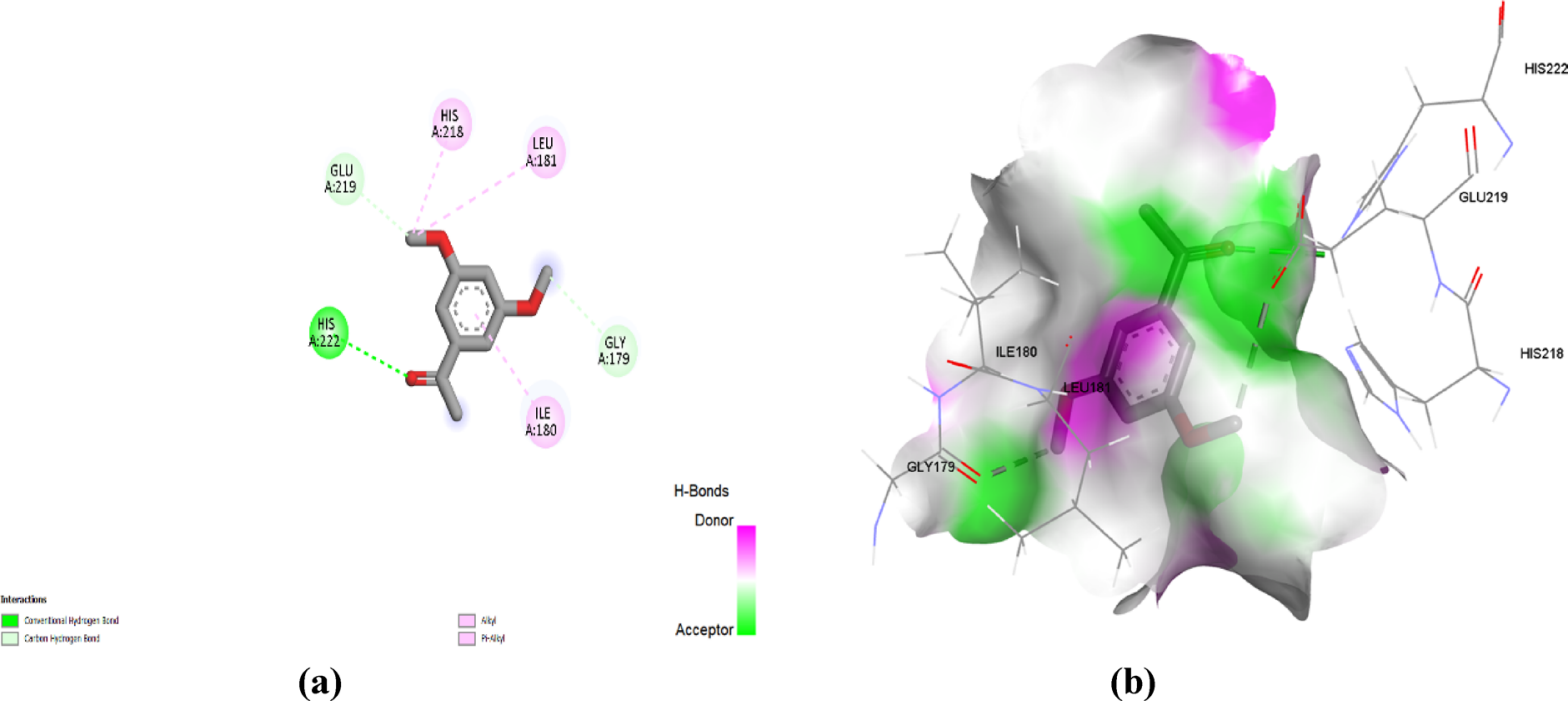
(**a**) Two-dimensional interactions and (**b**) three-dimensional interactions of 3’,5’-dimethoxyacetophenone with the active site of MMP12 (PDB ID: 2WO8).

**Fig. 8:**
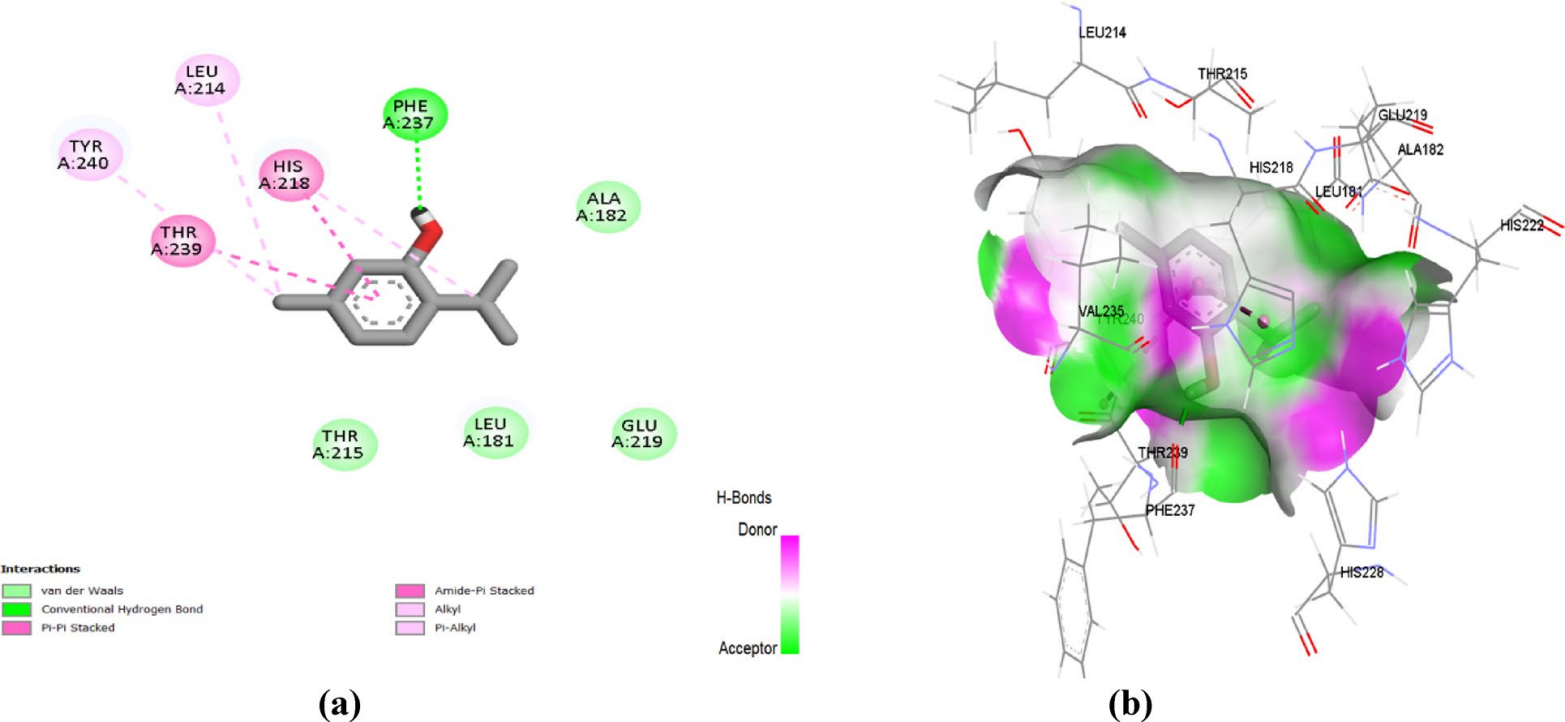
(**a**) Two-dimensional interactions and (**b**) three-dimensional interactions of thymol with the active site of MMP12 (PDB ID: 2WO8).

Regarding the docking simulation at MMP12, the 3’,5’-dimethoxyacetophenone and thymol obtained the highest docking score of ‒5.3 and ‒5.8 kcal/mol. The docked compounds depicted favoured anchoring at proximal distance from the zinc binding site with their polar functionality directed towards the polar lining residues. The interaction diagram (Fig. 7) showed that 3’,5’-Dimethoxyacetophenone interacted with five amino acid residues in the MMP12 active site. Specifically, the compound was bound to His222 through a conventional hydrogen bond that may contribute to its good affinity, besides carbon-hydrogen bonds to Glu219 and Gly179 residues. Both His222 and Glu219 belong to the fundamental residues mediating zinc-atom coordination (as part of His218, His222, and His228 triad) and acid/base functionality in the hydrolytic machinery, respectively^22^. The ability of 3’,5’-dimethoxyacetophenone to interact with these residues provides a rationale for prospective biological significance. In addition, alkyl and Pi-alkyl interactions were established with Ile180, His218, and Leu181. Moreover, thymol is involved in a conventional hydrogen bond with Phe237, together with hydrophobic interactions with His218, Thr239, Tyr240, and Leu214 residues, indicating the compound’s affinity to the MMP12 active site (Fig. 8). It is worth mentioning that the docked compounds predicted close-range hydrophobic contacts with key pocket residues, including Leu181 and Leu214, comprising the S1’ selectivity pocket of the MMK12 enzyme. This pocket is considered one of the most lipophilic and deepest known pockets suitable for anchoring the aromatic scaffolds of our docked compound, as well as being reported to have importance for driving selectivity towards MMK12 over other MMP family members^23,24^. Interactions with Leu181 and Leu214 have also been deposited with the coordinates of the MMP12’s co-crystalline ligand (‒8.1 kcal/mol) the thing which further confers the successful biological translation of the docking findings of both natural compounds.

**Fig. 9:**
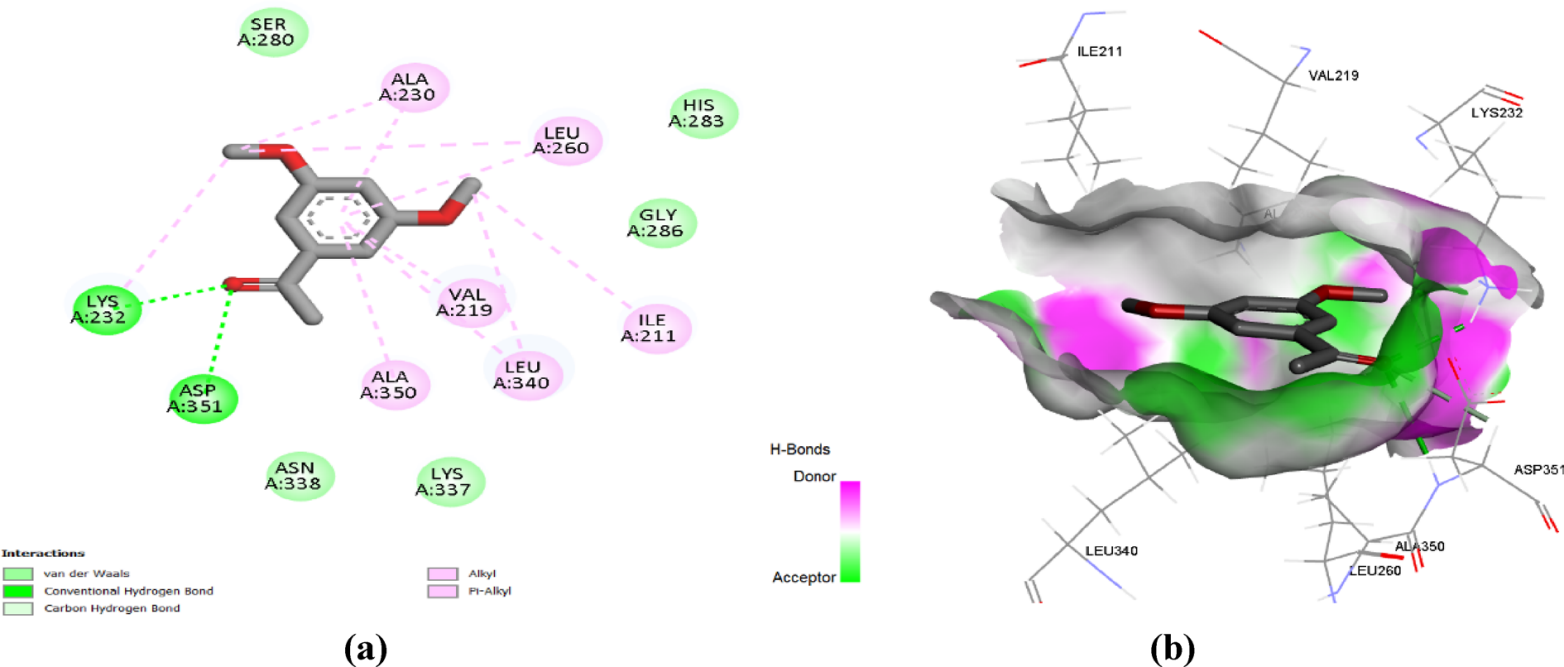
(**a**) Two-dimensional interactions and (**b**) three-dimensional interactions of 3’,5’-dimethoxyacetophenone with the active site of TGF-β (PDB ID: 6B8Y).

**Fig. 10:**
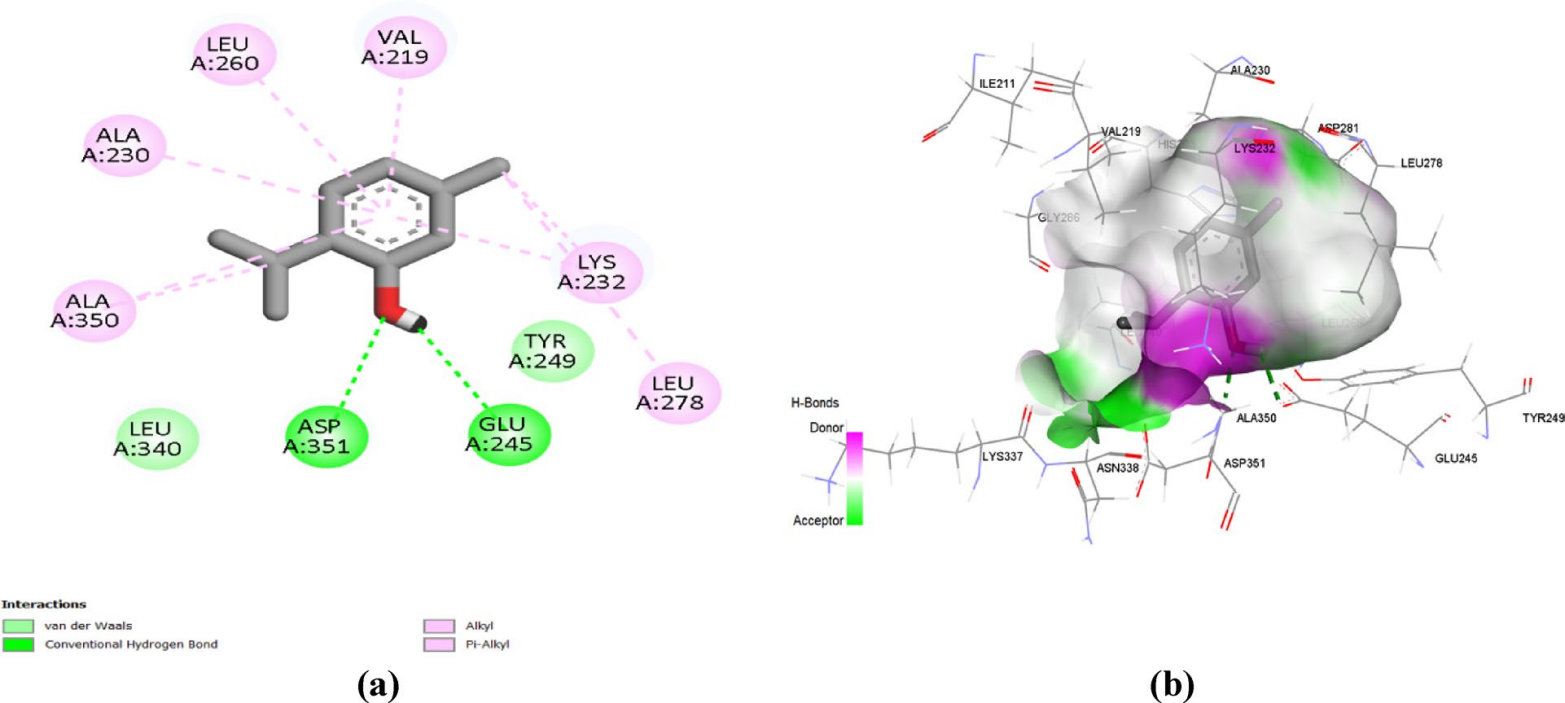
(**a**) Two-dimensional interactions and (**b**) three-dimensional interactions of thymol with the active site of TGF-β (PDB ID: 6B8Y).

Finally, both 3’,5’-dimethoxy acetophenone and thymol obtained the superior docking score of ‒ 5.2 and ‒ 5.7 kcal/mol, respectively, throughout their docking simulations at the TGF-β target. The docked natural compounds showed preferential anchoring at the ATP’s binding site of the TGF-β enzyme. Compounds depicted a particular settlement at the adenine-specific site (Lys232, Asp351, Glu245) and with relevant direction towards the hinge region residues (Asp281, Tyr282, His283). Figure 9 showed that 3′,5′-dimethoxyacetophenone interacted with Asp351 and Lys232 via conventional hydrogen bonds and with Ala230, Leu260, Ile211, Leu340, Val219, and Ala350 through alkyl and pi-alkyl interactions. While thymol is bound to the TGF-β active site via conventional hydrogen bonds with Asp351 and Glu245 and alkyl and pi-alkyl interactions with Leu278, Lys232, Val219, Leu260, Ala230, and Ala350 (Fig. 10).

Owing to the relatively smaller sizes of the natural compounds, these agents could not manage to mediate complete filling of the large pocket size, unlike the co-crystallized ligand that was assigned with higher docking score (‒ 6.2 kcal/mol). However, literature evidence highlights that adequate anchoring at the adenine pocket, which is the most energetically significant region of the kinase enzyme, can outcome ATP competition without sufficient filling of the entire cleft^25,26^. Further, our docked natural compounds showed optimum favored contacts with several kinase hotspot residues, including Glu245 as the conserved catalytic Lys–Glu salt-bridge region, as well as Asp351 of the activation loop DFG motif. Studies reported that relevant interactions towards several hotspot kinase residues can redeem sub-micromolar or nanomolar potency even at non-adequate pocket occupancy^27^. Finally, it has been reported that the kinase’s ATP pocket is quite flexible, where small ligands can be advantageous for minimizing the conformational strains as well as avoiding the entropic penalties commonly associated with binding with large-size ligands^28–30^. Thus, small molecules like dimethoxy acetophenone and thymol can suggest mediating relevant biological significance despite their small-sized architecture in relation to the co-crystallized ligand.

Based on the furnished docking findings for our docked compounds on the three targets, it has been evident that several identified metabolites are predicted with biological significance in wound healing, providing molecular mechanistic values. The latter was evident by the capability of each docked molecule to depict strong and biologically meaningful residue-wise contacts, being in concordance with mechanisms relevant to wound healing. These in silico outcomes postulate that multiple *Thymus* spp. Extract-derived metabolites can collectively contribute to the modulation of the key pathway elements involved in tissue repair. This would provide a mechanistic rationale in complement to the obtained in vitro activities. Thus, the presence of such bioactive metabolites within the Thymus spp. The extract would provide evidence that the experimentally recorded biological effects are developed from synergistic/cumulative contributions of different compounds rather than those of singular molecules. In brief, the integrated computational and in vitro findings highlight that the extract’s multi-modal activity reinforces the metabolites’ biological relevance through a molecular docking approach.

## Discussion

The present study provides a comprehensive comparative analysis of the chemical profiles and biological activities of EOs and *n*-hexane extracts from *Thymus vulgaris* and *Thymus serpyllum*, alongside a commercial thyme oil reference. The findings reveal significant chemotypic variations and distinct biological potentials, particularly highlighting the superiority of *T. serpyllum* EO in wound healing.

The GC-MS analysis confirmed the expected phytochemical diversity within the genus *Thymus*. The EO of *T. serpyllum* (TSV) was characterized as a thymol chemotype (74.47%), accompanied by significant levels of caryophyllene (9.89%), while the commercial reference oil was unexpectedly a carvacrol chemotype (80.37%). However, *T. vulgaris* EO (TVV) was also a thymol-rich chemotype (78.21%). *T. vulgaris* from Bosnia and Herzegovina was reported as a “thymol chemotype,” characterized by high levels of thymol (36.7%) and *p*-cymene (30.0%)^31^. In contrast, *T. serpyllum* from Montenegro was reported to contain *p*-cymene (19.04%) and geraniol (11.09%) as its primary constituents^32^. Such variations in chemical composition have a direct impact on the plant’s biological activity. This chemotypic variation is well-documented and is influenced by genetic factors, geographical origin, and environmental conditions^1^. Such differences in metabolite composition may reflect the influence of environmental and physiological conditions on secondary metabolite biosynthesis, as previously reported^33^, which emphasized that variations in phytochemical profiles directly affect the biological and pharmacological activities of plant-derived products.

The *n*-hexane extracts revealed a fundamentally different composition, dominated by long-chain aliphatic hydrocarbons and ethers, such as tritriacontane and hexatriacontane. The presence of thymoquinone, a compound with renowned antioxidant and anti-inflammatory properties, exclusively in the *n*-hexane extracts further underscores the value of non-polar fractions in phytochemical studies.

To provide a clearer chemotype differentiation between *T. vulgaris* and *T. serpyllum*, quantitative ratios of key constituents were calculated. *T. vulgaris* EO exhibited a markedly high thymol/carvacrol ratio of 6.36, consistent with a thymol-rich chemotype, whereas *T. serpyllum* showed a lower thymol/carvacrol ratio of 1.80, which reflects a mixed or carvacrol‑leaning profile. The comparison of compound classes showed that *T. vulgaris* contained a higher proportion of oxygenated monoterpenes relative to monoterpene hydrocarbons of a monoterpenes/sesquiterpenes ratio of 7.63. However, *T. serpyllum* demonstrated a more balanced distribution between monoterpenes and sesquiterpenes ratio of 2.75. These quantitative ratios facilitate a clear chemotypic distinction and highlight the differences in dominant chemical classes that contribute to the observed variations in biological activity.

The cytotoxicity assessment on human skin fibroblasts (HSF) was crucial for establishing a safety profile. Most samples exhibited low cytotoxicity (IC_50_ > 100 µg/mL). Crude plant extracts, IC_50_ values above 100 µg/mL are generally considered to indicate weak or no activity in most in vitro biological activities, including cytotoxic, antioxidant, antibacterial, and antiviral activities^34,35^. However, *T. serpyllum* EO (TSV) demonstrated potent cytotoxicity (IC_50_ = 18.48 µg/mL). This heightened toxicity can be attributed to its high concentration of phenolic monoterpenes, primarily thymol, which are known to disrupt cellular membranes^36^. This necessitates careful dose consideration, though the subsequent wound healing assays demonstrated its efficacy at a much lower, non-cytotoxic concentration (0.05 µg/mL). Thymol, *p*-cymene, and linalool were reported as the primary active anticancer constituents of *T. serpyllum* EO, with an IC_50_ value of 262 µg/mL, which provides a clear mechanistic insight linking the EO’s chemical composition to its anticancer potential^37^.

In the evaluation of antidiabetic potential, TSV was the only sample that showed moderate inhibitory activity against α-glucosidase (IC_50_ = 536.9 µg/mL), though it was significantly less potent than the standard drug acarbose. This activity was linked to its high phenolic content, as phenolics are known inhibitors of carbohydrate-digesting enzymes^17^. The lack of significant α-amylase inhibition suggests a more targeted mechanism for TSV.

The most compelling finding was the exceptional in vitro wound healing activity of TSV. At 0.05 µg/mL, TSV achieved complete wound closure in HSF cells within 72 h and outperformed all other samples. This profound effect on fibroblast migration suggests TSV promotes tissue regeneration by enhancing cellular motility and proliferation. The choice of the 0.05 µg/mL concentration for the wound healing assay was based on SRB cytotoxicity results, which showed that TSV exhibited significant cytotoxic effects only at much higher concentrations, with an IC_50_ value of 18.48 µg/mL. Thus, 0.05 µg/mL was selected as a safe, non-toxic concentration for assessing wound healing activity. To evaluate the statistical significance of the difference in toxicity between this wound-healing dose and higher, cytotoxic concentrations, a Two-Way ANOVA was performed using cytotoxicity percentages across varying doses of TSV and the reference drug Doxorubicin (Dox). The analysis revealed that TSV induced less than 5% toxicity at 0.05 µg/mL, while toxicity exceeded 98% at 100 µg/mL, with a highly significant increase in toxicity across concentrations (*p* < 0.0001). Post hoc analysis further confirmed that the toxicity observed at 0.05 µg/mL was statistically different from that at concentrations ≥ 10 µg/mL (*p* < 0.0001). These findings highlight a clear dose-dependent increase in cytotoxicity, supporting the safety of TSV at low doses and its potential therapeutic utility in promoting wound healing without inducing toxic effects. This biphasic response aligns with the concept of hormesis, where natural compounds exhibit beneficial effects at low concentrations and toxic effects at higher doses.

Recent studies have established a strong correlation between phenolic compounds and cytotoxic effects. *T. serpyllum* essential oil (EO) reported a significant cytotoxic activity against human cancer cell lines, with IC_50_ values ranging from 0.32 to 0.49 µL/mL^32^. In addition, Basal Cell Carcinoma (BCC) cancer stem cells demonstrated that *T. serpyllum* EO not only inhibited clonogenicity and cell migration but also suppressed critical oncogenic signaling pathways^37^. The high phenolic content of thymol and carvacrol in *Thymus* species plays a critical role in wound healing due to their well-documented antibacterial properties that help to maintain a sterile wound environment, thereby facilitating faster and more effective healing^38,39^. The combination of *T. vulgaris* honey and thymol-rich essential oil demonstrated superior effectiveness in burn treatment, resulting in the fastest healing and highest wound closure rates. This synergy highlights a promising natural approach to enhance wound repair^40^.

To rationalize this activity, molecular docking studies were conducted on the major constituents of TSV against key wound healing targets: collagenase, MMP-12, and TGF-β. The results indicated strong binding affinities for thymol and 3’,5’-dimethoxyacetophenone. These compounds formed stable complexes through hydrogen bonds and hydrophobic interactions. The inhibition of collagenase and MMP-12, which can impede healing if overexpressed, would facilitate tissue repair^11^. Simultaneously, interaction with TGF-β, a master regulator of cell proliferation, could further stimulate the healing cascade. The synergistic action of these metabolites likely contributes to the observed potent wound healing effect of the whole oil.

## Conclusion

This study revealed chemotypic with synergistic metabolite interactions and biological differences between *Thymus vulgaris* and *Thymus serpyllum*. *T. serpyllum* EO showed the strongest wound-healing activity at low, non-cytotoxic concentrations, alongside moderate α-glucosidase inhibition. Its dose-dependent dual effect—cytotoxic at high doses yet regenerative at low doses—was supported by in silico docking. Molecular docking simulation highlighted relevant binding affinity for the major metabolites of *T. serpyllum* extract. Metabolites, including thymol, 3’,5’-Dimethoxy acetophenone, caryophyllene, and α-Selinene, predicted relevant interactions and pocket accommodation at the orthosteric pockets of three key proteins associated with wound healing. As these metabolites coexist simultaneously in the extract, this suggests that these major metabolites would collectively and additively produce the synergistic wound healing effect obtained for the extract. The metabolites’ combined actions were deemed strong, as evidenced by the acceptable docking scores of all major identified compounds. These promising activities are in line with other Lamiaceae species; however, *Thymus* spp. exhibits a broader spectrum of bioactivity driven by its variable thymol/carvacrol content. These findings highlight the therapeutic potential of *T. serpyllum* EO and emphasize the value of chemotypic standardization to ensure consistent performance in wound-related and metabolic applications.

## Supplementary Information

Below is the link to the electronic supplementary material.

Supplementary Material 1

## Data Availability

The data that support the findings of this study are available from the authors upon reasonable request. For your request, please contact Dr. Mohamed M.M. Abdelrazek ( [mohamed.abdelrazek@buc.edu.eg](mailto: mohamed.abdelrazek@buc.edu.eg) ).
