# Supplementary material for: Comparative chemical and biological study of essential oils and n-hexane extracts of Thymus vulgaris and Thymus serpyllum (Lamiaceae)
Source: Sci Rep. 2026 Jan 16;16:2615. doi: 10.1038/s41598-025-33660-w (PMC12819475; doi:10.1038/s41598-025-33660-w)
Supplement: Supplementary file 1 — Supplementary Material 1 [file 41598_2025_33660_MOESM1_ESM.docx]

**Supplementary Data**

**Comparative Chemical and Biological Study of Essential Oils and *n*-Hexane Extracts of *Thymus vulgaris* and *Thymus serpyllum* (Lamiaceae)**

Mohamed M.M. AbdelRazek^1*^, Asmaa M. Atta^2^, Nariman H. Kandil^1^, Iriny E. G. Girgis^3^, Rana T. O. Elsayed^3^, Bassant R. M. Abdel-Latif^3^, Asmaa E. Abdel-Halim^3^, Shada G. I. Salama^3^, Tag El-Din M. Ahmed^3^, Mennat Allah S. AbdelRazek^3^, Fady H. Foad^3^, Rodaina M. S. Elsayed^3^, Sara H . E . Aslan ^3^, Nada I. E . Badawy^3^, Engy A.Farouk^1^, Sara A. Omran^1^, Khaled M. Darwish^4,5^, Safaa A. El-Moghazy^1^

^1^ Department of Pharmacognosy, Faculty of Pharmacy, Badr University in Cairo (BUC), Cairo 11829, Egypt.

^2^ Department of Pharmaceutical Chemistry, Faculty of Pharmacy, Badr University in Cairo (BUC), Badr, Cairo 11829, Egypt.

^3^ Internship Researcher, Faculty of Pharmacy, Badr University in Cairo (BUC), Cairo 11829, Egypt.

^4^ Department of Medicinal Chemistry, Faculty of Pharmacy, Galala University, New Galala 43713, Egypt

^5^ Medicinal Chemistry Department, Faculty of Pharmacy, Suez Canal University, Ismailia 41522, Egypt

^*^ Corresponding Author: [mohamed.abdelrazek@buc.edu.eg](mailto:mohamed.abdelrazek@buc.edu.eg)

**Index**

[Figure S 1: 2D Interaction of the three co-crystallized ligands with A) Collagenase (PDB ID: 5O7E), B) MMP12 (PDB ID: 2WO8) and C) TFG-ß (PDB ID: 6B8Y). 2](#_Toc206064000)

[Figure S 2: Docking validation of the three targets: A) Collagenase (PDB ID: 5O7E), B) MMP12 (PDB ID: 2WO8) and C) TFG-ß (PDB ID: 6B8Y). 3](#_Toc206064001)

**
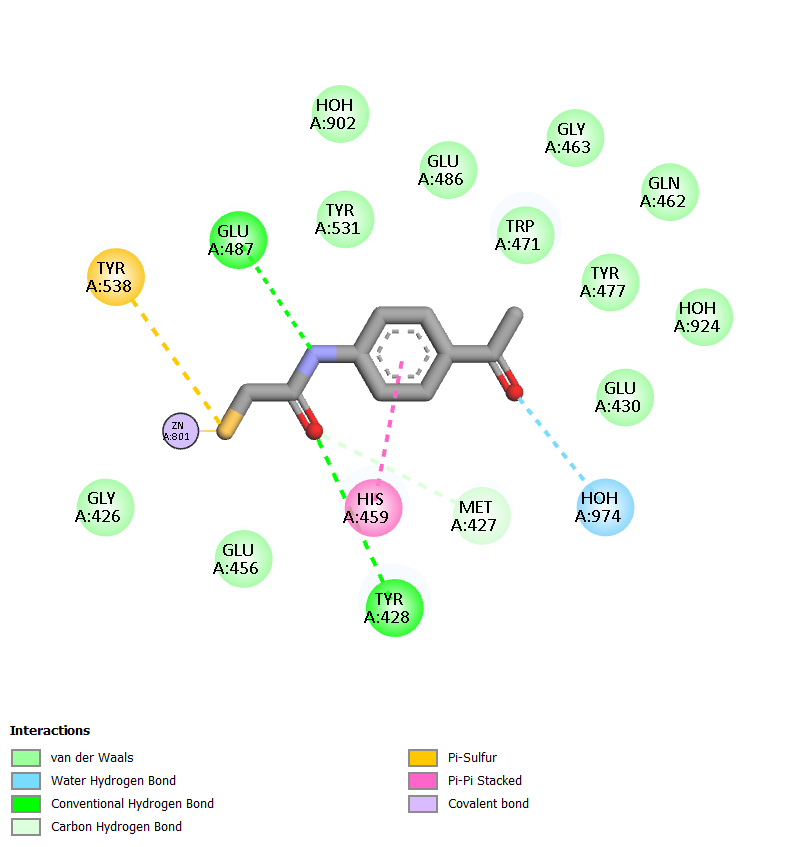
**

**(A)**


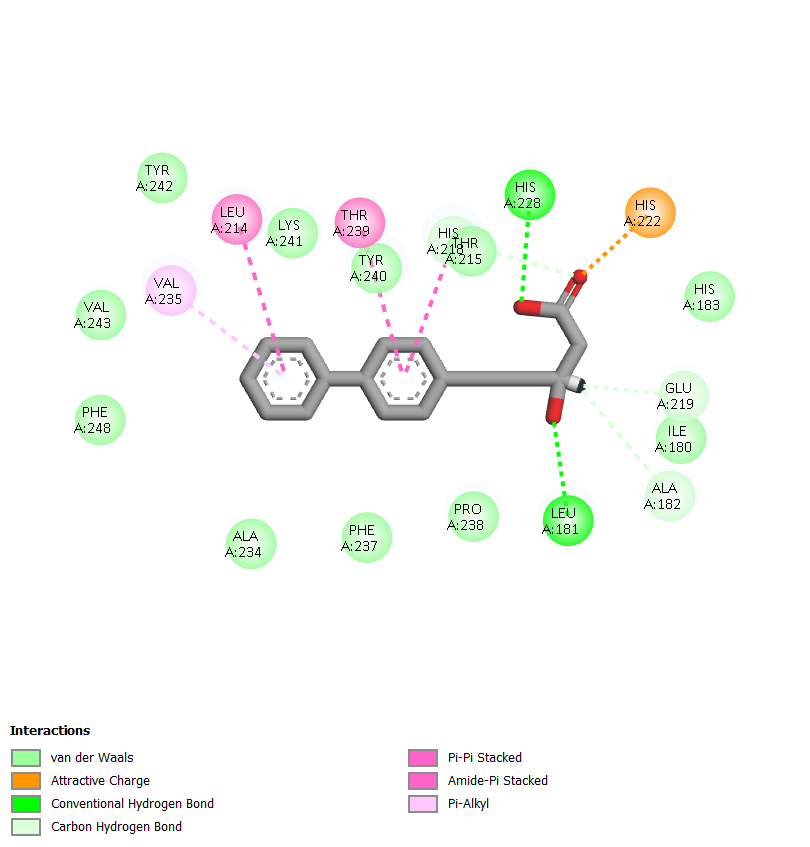


**(B)**

**
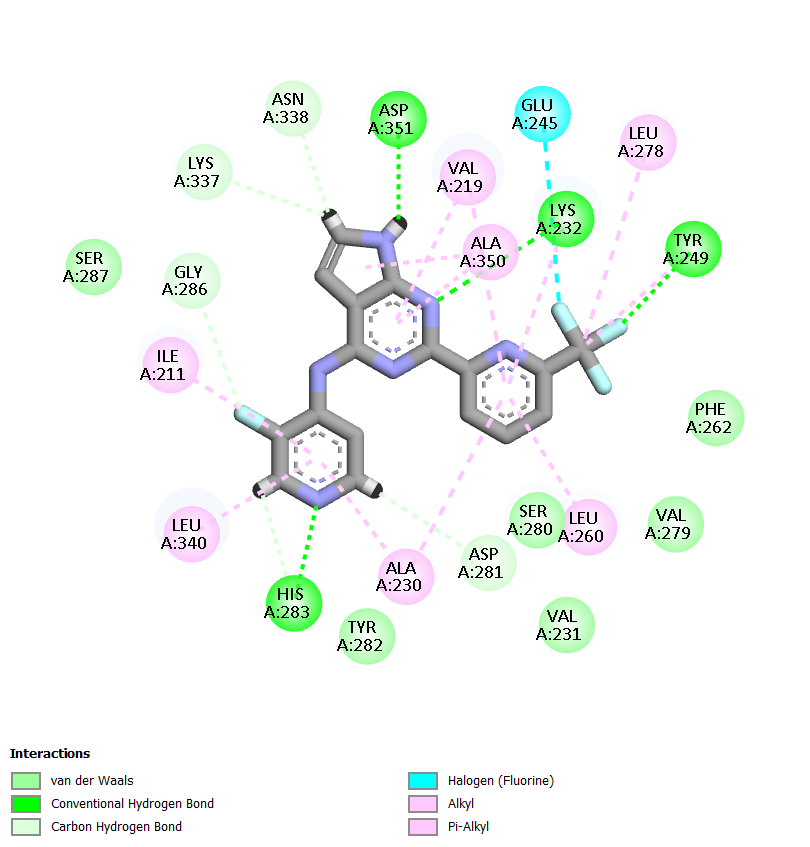
**

**(C)**

Figure S 1: 2D Interaction of the three co-crystallized ligands with (A) Collagenase (PDB ID: 5O7E), (B) MMP12 (PDB ID: 2WO8) and (C) TFG-ß (PDB ID: 6B8Y).


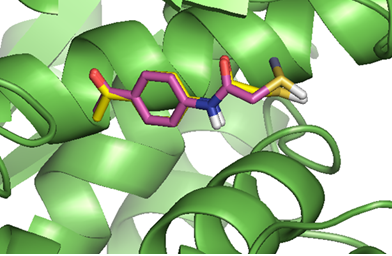


(A)


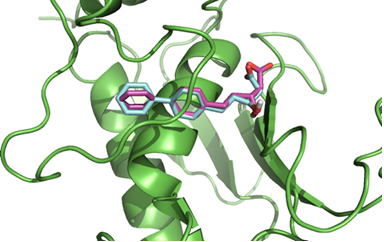


(B)


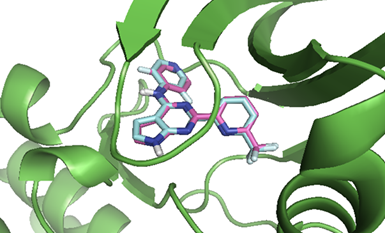


(C)

Figure S 2: Docking validation of the three targets: A) Collagenase (PDB ID: 5O7E), B) MMP12 (PDB ID: 2WO8) and C) TFG-ß (PDB ID: 6B8Y).
